# Lnc-RPS6P3 Inhibits Influenza A Virus Replication and Attenuates the Inhibitory Effect of NS1 on Innate Immune Response

**DOI:** 10.3390/microorganisms12040654

**Published:** 2024-03-26

**Authors:** Mingge Wang, Xinli Yao, Xiaomei Tong, Dandan Qi, Xin Ye

**Affiliations:** 1 School of Life Sciences, Division of Life Sciences and Medicine, University of Science and Technology of China, Hefei 230027, China; mgwang@mail.ustc.edu.cn; 2 Key Laboratory of Pathogenic Microbiology and Immunology, Institute of Microbiology, Chinese Academy of Sciences (CAS), Beijing 100101, China; 17863934700@163.com (X.Y.); cocoa3333@126.com (X.T.); qiqiwhu@126.com (D.Q.); 3 Savaid Medical School, University of Chinese Academy of Sciences, Beijing 100049, China

**Keywords:** lnc-RPS6P3, influenza A virus, NP, NS1, vRNP, antiviral immunity

## Abstract

Host factors play important roles in influenza A virus (IAV) replication. In order to identify novel host factors involved in IAV replication, we compared the differentially expressed genes in A549 cells after IAV infection. We found that lncRNA lnc-RPS6P3 was up-regulated upon viral infection and poly(I:C) and IFN-β treatment, indicating it was an interferon-stimulated gene. Functional analysis demonstrated that overexpression of lnc-RPS6P3 inhibited IAV replication while knockdown of lnc-RPS6P3 promoted viral infection in A549 cells. Lnc-RPS6P3 inhibited both transcription and replication of IAV. Further study showed that lnc-RPS6P3 interacted with viral NP and interfered with NP self-oligomerization and, consequently, inhibited vRNP activity. In addition, lnc-RPS6P3 interacted with viral NS1 and reduced the interaction of NS1 and RIG-I; it also attenuated the inhibitory effect of NS1 on IFN-β stimulation. In conclusion, we revealed that lnc-RPS6P3 is an interferon-stimulated gene that inhibits IAV replication and attenuates the inhibitory effect of NS1 on innate immune response.

## 1. Introduction

Influenza A viruses cause contagious respiratory infections that can lead to mild to severe illness and even death in high-risk populations. Four influenza pandemic outbreaks occurred in 1918, 1957, 1968 and 2009, and the 1918 “Spanish” flu caused 50 million deaths worldwide [1,2]. WHO reported that influenza caused about 30,000–500,000 deaths annually [1]. Influenza A virus can infect a wide range of hosts, including humans, birds and pigs. Due to the cross-species transmission, animal influenza viruses such as the avian influenza A viruses H5N1 and H7N9 can infect humans and have a high fatality rate. Once the viruses gain the ability of human-to-human transmission, they might cause severe public health problems. Vaccines and antiviral drugs were developed to control influenza infections. Influenza A virus (IAV) belongs to the family Orthomyxoviridae, which consists of eight segments of negative single-stranded RNA encoding 10 essential proteins for viral replication. PB1, PB2 and PA form a heterotrimeric polymerase complex that is required for both viral RNA replication and transcription [3,4]. It is known that vRNP activity is required for both viral transcription and replication [5,6]. The tail loop of the nucleoprotein (aa 402–428) is crucial for oligomerization and ribonucleoprotein activities [7,8,9]. To be efficiently replicated, influenza viruses need to evade the host antiviral immune response. The NS1 protein is the key factor required for the virus to interfere with host innate immune responses via multiple pathways [10,11,12]. For example, NS1 interacts with retinoic acid-inducible protein I (RIG-I) and inhibits RIG-I triggered downstream signaling pathways and the expression of type I interferon [13,14,15]. NS1 binds to E3 ubiquitin ligase TRIM25 to block RIG-I ubiquination and dimerization, which consequently inhibits the transcription of IFN-β [16,17].

Host factors have been found to be involved in regulating influenza A virus replication [18,19,20]. The IFN-β-stimulated gene IFITM3 can inhibit influenza A replication at an early stage [21]. MxA can effectively block highly pathogenic influenza A virus replication [22,23]. Currently, more and more studies are reporting that non-coding RNAs (lncRNA, circ-RNA) affect IAV replication in both positive and negative manners [24]. LncRNA-155 suppresses IAV replication by activating host antiviral innate immunity [25]. Lnc-ISG20 enhances ISG20 expression and inhibits influenza A virus replication [26]. CircVAMP3 interferes with vRNP complex activity by interacting with NP and promotes the activation of IFN-β by alleviating the inhibitory effect of NS1 to RIG-I, and it eventually restricts influenza A virus replication [27]. However, lncRNA NRAV and lnc-MxA significantly promote influenza A virus replication and virulence by reducing IFN-β production [28,29]. These studies demonstrate that non-coding RNAs engage in complex functions during viral infection.

In this study, we focused on the function and mechanism of lncRNA lnc-RPS6P3 in regulating IAV replication. We found that lnc-RPS6P3 inhibits the replication of IAV. Further studies indicate that lnc-RPS6P3 interacts with NP and inhibits its oligomerization and consequently suppresses vRNP activity. Lnc-RPS6P3 binds to NS1 to interfere with its binding with RIG-I and therefore attenuates its inhibitory effect on the activation of the antiviral immune response. Our studies reveal that lnc-RPS6P3 acts as a restriction factor on IAV replication via inhibiting viral replication and regulating the antiviral immune response.

## 2. Materials and Methods

### 2.1. Cell Line and Viruses

A549, HEK293T and MDCK cell lines were obtained from the China Infrastructure of Cell Line Resources and cultured in Dulbecco’s modified Eagle’s medium (DMEM, GIBCO, Waltham, MA, USA). All cell cultures were supplemented with 10% fetal bovine serum (FBS, GIBCO) and maintained at 37 °C in a humidified incubator with 5% CO_2_. HEK293T-IAV-Luc cells were generated by transfection of plasmid pREP-FluA-Luc (kindly provided by Prof. Andrew Pekosz, Johns Hopkins University, Baltimore, MD, USA) where the firefly reporter gene is right behind the NP promoter of influenza virus and which expresses firefly luciferase upon influenza A virus infection. lncRPS6P3-konckdown A549 cells were generated using shRNA. The influenza A virus strains A/WSN/33 (A/WSN; H1N1), A/Puerto Rico/8/34 (A/PR8; H1N1) and A/California/2009/04 (A/CA04; H1N1) (kindly provided by Prof. Wenjun Liu, Institute of Microbiology, Chinese Academy of Sciences, Beijing, China) were propagated in MDCK cells.

### 2.2. Plasmids and Antibodies

The full-length sequence of human lnc-RPS6P3 was cloned into the pLentiLox3.7 plasmid. To generate pLL3.7-lnc-RPS6P3-S1, the S1 aptamer sequence containing a 44-nt (5′-ACCGACCAGAATCATGCAAGTGCGTAAGATAGTCGCGGGCCGGG-3′) was inserted into the 3′-end of lnc-RPS6P3. The short hairpin RNA (shRNA) targeting lnc-RPS6P3 was constructed into pSIH-H1-GFP lentiviral vector. The shRNA target sequences were as follows: shRNA-lnc-RPS6P3#1, GCGTATTGCTCTGAAGAAACA; shRNA-lnc-RPS6P3#2, GGCGTATTGCTCTGAAGAAAC.

Mouse anti-M1, anti-PB1, anti-PB2, anti-PA and rabbit anti-NP antibodies were kindly provided by Prof. Wenjun Liu. Mouse anti-β-actin (KM9001), anti-GAPDH (KM9002) antibodies were purchased from Sungene Biotech Co. (Tianjin, China). Mouse anti-Flag antibody (F3165) was purchased from Sigma (Kawasaki, Japan). Mouse anti-Myc antibody (sc-40) was purchased from Santa Cruz (Dallas, TX, USA). HRP-conjugated secondary antibodies (115-035-003 (anti-mouse IgG) or 111-035-003 (anti-rabbit IgG)) were purchased from The Jackson Laboratory (Bar Harbor, ME, USA). Rabbit anti-RIG-I antibody (3743S) was purchased from Cell Signaling Technology (Danvers, MA, USA).

The protein-coding potential of lnc-RPS6P3 was analyzed using the Coding Potential Calculator (CPC2.0) software (http://cpc2.gao-lab.org/index.php) accessed on 5 June 2022 [30].

### 2.3. Cell Transfection and Lentiviral Production

For transient transfection, cells were plated 12–24 h prior to transfection to achieve 50–70% confluency and then transfected with Hieff Trans (Yeasen, Shanghai, China) or Vigofect reagent (Vigorous Biotechnology, Beijing, China) according to the manufacturer’s protocol. Cells were collected 48 h after transfection.

For generating lentivirus, HEK 293T cells were transfected with pLL-3.7-lnc-RPS6P3 or pSIH-lnc-RPS6P3-shRNA and the packaging plasmids (pLP1, pLP2, and pLP/VSVG) for 48 h. Then the supernatant was collected, followed by filtration with 0.45-μm sterile filters (Merck Millipore, Burlington, MA, USA). For generating stable cell lines, A549 cells were infected with indicated lentiviruses for 12 h. The GFP positive cells were selected by flow cytometry.

### 2.4. Immunoblotting

Cells were harvested and lysed in lysis buffer containing 10% Glycerin, 150 mM NaCl, 1% Triton X-100, 1 mM EDTA (pH 8.0), 20 mM Hepes (pH 7.5) supplemented with protease inhibitor (Roche, Basel, Switzerland). Whole-cell lysates were centrifuged at 12,000 rpm at 4 °C for 15 min. After centrifugation, the supernatant was collected, and protein concentration was determined using BCA Protein Assay Kit (DINGGUO, Beijing, China). Then the samples were subjected to immunoblotting with the indicated primary antibodies followed by HRP-conjugated anti-rabbit IgG or anti-mouse IgG secondary antibodies. The intensity of detected proteins was quantified by Image J 1.8.0.

### 2.5. RNA Isolation and Quantitative RT-PCR (RT-qPCR)

Total RNA from cells was extracted using Trizol (Invitrogen, Waltham, MA, USA). The equal amounts of RNA were reverse-transcribed using Hifair II 1st Strand cDNA Synthesis SuperMix (Yeasen). Quantitative real-time PCR was performed using Hieff qPCR SYBR Green Master Mix (Yeasen) with GAPDH as an internal control. The relative amount of RNAs in lnc-RPS6P3-expressing cells or lnc-RPS6P3-knockdown cells was calculated compared to control cells. Primer sequences are provided as follows:

lnc-RPS6P3-F: 5′-TCAGCGACAGGAACGACAAA-3′, lnc-RPS6P3-R: 5′-CTGCAAACAGCTTCGTGCTT-3′; IFN-β-F: 5′-AACAAGTGTCTCCTCCAAATTGC-3′, IFN-β-R: 5′-GCAGTATTCAAGCCTCCCATTC-3′; M1-F: 5′-TCTGATCCTCTCGTCATTGCAGCAA-3′, M1-R: 5′-AATGACCATCGTCAACATCCACAGC-3′; NP-F: 5′-TGGCACTCCAATTTGAATGATG-3′, NP-R: 5′-TCCATTCCTGTGCGAACAAG-3′; GAPDH-F: 5′-GTCGGAGTCAACGGATTTGG-3′, GAPDH-R: 5′-CGCCCCACTTGATTTTGG-3′.

### 2.6. Luciferase Reporter Assay

HEK293T cells were seeded in 24-well plates and co-transfected with pLL3.7-lnc-RPS6P3, pGL4-IFN-β-luc and pRL-TK (Renilla, Quincy, IL, USA) as the internal control for 24 h. The cell lysates were harvested for the firefly and Renilla luciferase assays. HEK293T cells were co-transfected with pcDNA-PB2, pcDNA-PB1, pcDNA-PA, pCAGGS-NP, pHH21-vNS-Luc and pLL3.7-lnc-RPS6P3, and with pRL-TK (Renilla) as the internal control, for 36 h. Cell lysates were collected to measure the firefly and Renilla luciferase activities. The relative luciferase activity was normalized to Renilla luciferase activity.

For NanoLuc luciferase assays [31], LgBiT-NP and NP-SmBiT fusion protein-expressing plasmids were generated, in which protein NP was fused to the C-terminus of large NanoLuc fragment (pLenti-EF1a-LgBiT-NP) and the N-terminus of small NanoLuc fragment (pCDH-NP-SmBiT), respectively. HEK293T cells were co-transfected with pLL3.7-lnc-RPS6P3, pLenti-EF1a-LgBiT-NP and pCDH-NP-SmBiT in 24-well plates and harvested 24 h post-transfection. NanoLuc luciferase activity was measured using the Nano-Glo^®^Live Cell Assay System (Promega, Madison, WI, USA) according to the manufacturer’s protocol.

### 2.7. Co-Immunoprecipitation (Co-IP) and RNA Immunoprecipitation (RIP)

Cells were lysed in lysis buffer (10% Glycerin, 150 mM NaCl, 1% Triton X-100, 1 mM EDTA (pH 8.0), 20 mM Hepes (pH 7.5), 1 mM PMSF) supplemented with protease inhibitor (Roche) and RNase inhibitor (ThermoFisher Scientific, Waltham, MA, USA) at 4 °C. The lysates were incubated with anti-Flag M2 affinity gel (Sigma) or anti-Myc agarose (Shanghai Genomics Technology, Shanghai, China) at 4 °C for 4 h. For anti-NP immunoprecipitation, the lysates were incubated with anti-NP antibody or control anti-IgG and protein A beads overnight at 4 °C. The immunoprecipitates were washed with lysis buffer and then resolved in SDS loading buffer and subjected to immunoblotting with indicated antibodies. For RIP experiments, lysates were collected by centrifugation at 12,000 rpm for 15 min at 4 °C, followed by incubation with anti-Flag M2 affinity gel or anti-Myc agarose at 4 °C for 4 h. Then the samples were washed five times in lysis buffer. RNA samples were collected from cell lysates and the immunoprecipitates, extracted with 1ml Trizol and subjected to RT-qPCR analysis.

### 2.8. RNA Pulldown Assay and Mass Spectrometry Analysis

The cells were lysed with ice-cold lysis buffer (10 mM Tris-HCl (pH 7.5), 150 mM NaCl, 10 mM EDTA (pH 8.0), 0.5% Triton X-100, 1 mM PMSF, 1 mM DTT) containing protease inhibitor (Roche) and RNase inhibitor (Thermo Fisher Scientific). The cell lysates were incubated with streptavidin T1 magnetic beads (Invitrogen) at 4 °C for 4 h. Then, the beads were washed with the washing buffer (10 mM Tris-HCl (pH 7.5), 10 mM NaCl, 10 mM EDTA (pH 8.0), 0.5% Triton X-100). The RNA-associated proteins were dissociated from the beads and detected by immunoblotting. For RNA–protein interaction in vitro assay, S1-labeled lncRNA-RPS6P3 or S1-labeled antisense lncRNA-RPS6P3 were in vitro transcribed using a MEGAscript T7 transcription Kit (Invitrogen) and then incubated with purified proteins as indicated. Then, Streptavidin T1 magnetic beads were added and incubated at 4 °C overnight. The RNA pulldowned samples were subjected to mass spectrometry. The data were analyzed by performing a MASCOT search against the SWISS-PROT human database and IAV database.

### 2.9. Plaque Assay

MDCK cells were seeded in 12-well plates and incubated with serial dilutions of virus samples in serum-free DMEM medium supplemented with 4 μg/mL of TPCK-trypsin for 1 h. Cells were then overlaid with DMEM containing 1% low melting point agarose (Bio-Rad, Hercules, CA, USA) and 2 μg/mL of TPCK-trypsin, and the plates were settled at 4 °C for 10 min and incubated at 37 °C for 2–3 days. Then, the cells were fixed with 4% formaldehyde and stained with 0.1% crystal violet. The visible plaques were counted, and viral titers were calculated.

### 2.10. RNA Fluorescence In Situ Hybridization (FISH)

A549 cells were seeded on coverslide in 24-well plate. The cells were fixed with 4% paraformaldehyde at room temperature for 15 min and permeabilized with PBS containing 1% Triton X-100 for 15 min. Then, cells were blocked with hybridization buffer (50% formamide, 300 mM NaCl, 300 mM Sodium citrate) at 42 °C for 30 min, followed by hybridization with the biotin-labeled lnc-RPS6P3 probe at 42 °C overnight. The nuclei were stained with DAPI. The cells on slides were imaged under confocal fluorescence microscope. A biotin-labeled oligonucleotide probe (AAATGTTTCTTCAGAGCAATACGC) for lnc-RPS6P3 was synthesized by BGI Tech Solutions Co., Ltd. (Hong Kong, China).

### 2.11. Cell Fractionation

The cells were harvested and fractionated into nuclear and cytoplasmic fractions with nuclear and cytoplasmic extraction reagents (Applygen, Beijing, China) according to the manufacturer’s instructions. RNA was extracted from each fraction and subjected to qPCR. GAPDH mRNA was used as a cytoplasmic marker, and lncRNA MALAT1 was used as a nuclear marker.

### 2.12. Statistical Analysis

All statistical analyses were performed using the GraphPad Prism 10.1.1 Software. Student’s *t*-test was used for two-group comparisons, and two-way analysis of variance (ANOVA) was used for multiple-group comparisons. Data are presented as the means ± standard deviation (SD). ns, not significant. *p* < 0.05 was considered significant.

## 3. Results

### 3.1. Lnc-RPS6P3 Is Induced upon IAV Infection and IFN-β Treatment

**Figure 1:**
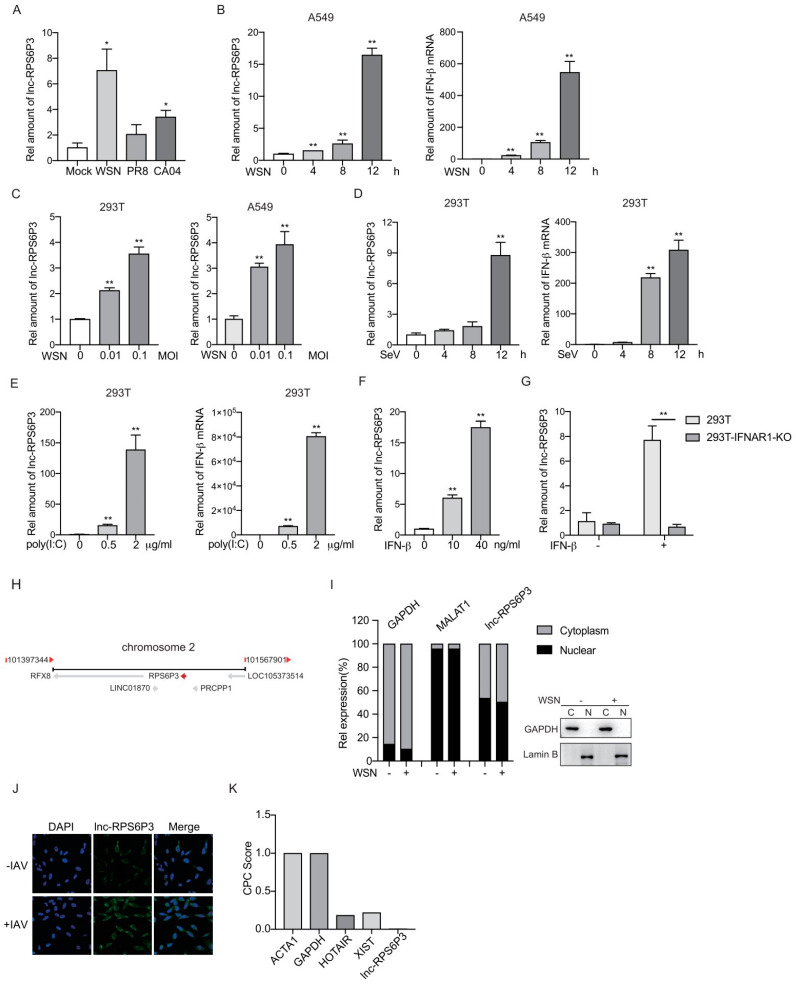
Lnc-RPS6P3 is induced upon IAV infection and IFN-β treatment. (**A**,**B**) A549 cells were infected with WSN, PR8 or CA04 at an MOI of 1 for 8 h (**A**) or infected with WSN at an MOI of 1 for indicated time (**B**). The amount of lnc-RPS6P3 and IFN-β mRNA were measured by RT-qPCR. (**C**) 293T and A549 cells were infected with WSN at different MOI for 12 h. The amount of lnc-RPS6P3 was measured by RT-qPCR. (**D**,**E**) 293T cells were infected with Sendai virus (SeV) for indicated time (**D**) or transfected with poly(I:C) for 8 h (**E**). The amounts of lnc-RPS6P3 and IFN-β mRNA were measured by RT-qPCR. (**F**,**G**) 293T cells (**F**) and 293T IFNAR1 KO cells (**G**) were treated with IFN-β for 12 h. The amount of lnc-RPS6P3 was measured by RT-qPCR. (**H**) The locus of lnc-RPS6P3 on chromosome. (**I**) 293T cells were infected without or with WSN at an MOI of 0.1 for 12 h. The RNAs (**left panel**) and proteins (**right panel**) from cytoplasm and nucleus were extracted and subjected to RT-qPCR and immunoblotting, respectively. (**J**) A549 cells were infected with A/WSN/33 at an MOI of 0.5 for 8 h, fixed and subjected to RNA FISH with a biotin-labeled lnc-RPS6P3 probe (green). Nuclei were stained with DAPI (blue). (**K**) The coding probability of lnc-RPS6P3 was estimated by CPC2.0 algorithms, with ACTA1 and GAPDH as coding RNA controls and HOTAIR and XIST as noncoding RNA controls. Data are shown as the mean ± SD. * *p* < 0.05 and ** *p* < 0.01.

In order to identify host factors involved in influenza A virus replication, we analyzed the differentially expressed lncRNAs in influenza A virus-infected A549 cells and identified a set of lncRNAs involved in viral replication. In this study, we focused on lncRNA RPS6P3 (NONCODE ID: NONHSAT072829), which is upregulated upon infection with the influenza A virus (Figure 1A–C). It can also be induced by a Sendai virus infection and poly(I:C) treatment (Figure 1D,E). In addition, lnc-RPS6P3 was significantly increased in IFN-β-treated 293T cells but not in IFN-β receptor IFNAR1 knockouted 293T cells, suggesting that it is an IFN-β-stimulated gene (Figure 1F,G). Lnc-RPS6P3 is located on chromosome 2 (Figure 1H). We examined the subcellular localization of lnc-RPS6P3 by cell fractionation and RT-qPCR. The data showed that lnc-RPS6P3 was localized in both the nucleus and cytoplasm, and there was no subcellular localization difference between cells infected with WSN and control cells (Figure 1I). RNA fluorescence in situ hybridization (FISH) confirmed that lnc-RPS6P3 was localized in both the nucleus and cytoplasm and notably induced upon infection with the influenza A virus (Figure 1J). Then, we analyzed the protein-coding potential of lnc-RPS6P3 using the Coding Potential Calculator (CPC2.0) software. ACTA1 and GAPDH served as coding RNA controls, while HOTAIR and XIST served as noncoding RNA controls. The predicting result indicated that lnc-RPS6P3 lacks the coding capability (Figure 1K).

### 3.2. Lnc-RPS6P3 Inhibits the Replication of Influenza A Virus

**Figure 2:**
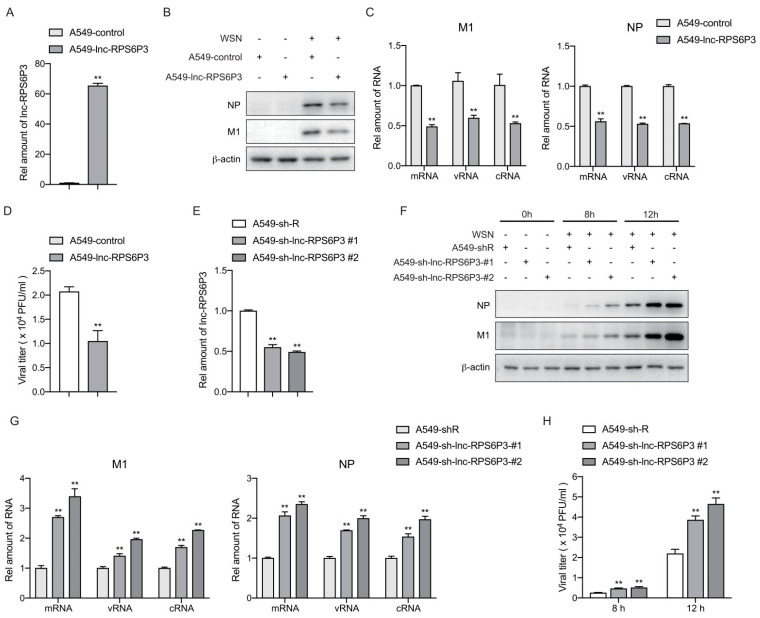
Lnc-RPS6P3 inhibits the replication of influenza A virus. (**A**–**D**) A549 cells were infected with lentivirus-expressing lnc-RPS6P3 or control lentivirus. The amounts of lnc-RPS6P3 in lnc-RPS6P3-expressing A549 cells and control cells were determined by RT-qPCR, using GAPDH as internal control (**A**). The cells were then infected with WSN at MOI of 0.1 for 12 h. The cell lysates were harvested for immunoblotting with indicated antibodies (**B**). The amount of mRNA, vRNA and cRNA of M1 and NP were measured by RT-qPCR (**C**). The supernatants were collected for measuring the viral titer by plaque assay (**D**). (**E**–**H**) A549 cells were infected with lentivirus carrying shRNA-targeting lnc-RPS6P3 (sh-lnc-RPS6P3#1, sh-lnc-RPS6P3#2) or control lentivirus (sh-R). The relative amount of lnc-RPS6P3 was determined by RT-qPCR (**E**). The cells were infected with WSN at MOI of 0.1 for 8 h and 12 h. The cell lysates were harvested for immunoblotting with indicated antibodies (**F**). The amount of mRNA, vRNA and cRNA of M1 and NP were measured by RT-qPCR (**G**). The supernatants were collected for measuring the viral titer by plaque assay (**H**). Data are shown as the mean ± SD. ** *p* < 0.01.

Next, we analyzed whether lnc-RPS6P3 can affect the replication of the influenza A virus. We generated lnc-RPS6P3-stably overexpressing A549 cells, named A549-lnc-RPS6P3 and A549-control cells. The relative amount of lnc-RPS6P3 was measured by RT-qPCR (Figure 2A). Then, the cells were infected with WSN at MOI of 0.1 for 12 h. The viral proteins of M1 and NP in the cell lysates were determined by immunoblotting. The data showed that the level of M1 and NP proteins was lower in lnc-RPS6P3-overexpressing cells than in control cells (Figure 2B). Meanwhile, the RT-qPCR data showed that the levels of mRNA, vRNA and cRNA of M1 and NP were significantly lower in lnc-RPS6P3-overexpressing cells than in control cells (Figure 2C). The supernatants were collected for measuring the viral titer by plaque assay. The result demonstrated that the viral titer was significantly decreased in the supernatant from lnc-RPS6P3-overexpressing cells (Figure 2D). We then performed similar experiments in lnc-RPS6P3 knockdown cells (A549-sh-lnc-RPS6P3-#1, A549-sh-lnc-RPS6P3-#2) and found that both the protein and RNA (mRNA, vRNA and cRNA) levels of M1 and NP in cells and the viral titer in the supernatant were obviously higher in lnc-RPS6P3 knockdown cells than in control cells (Figure 2E–H). These data demonstrated that lnc-RPS6P3 inhibits the replication of the influenza A virus.

### 3.3. Lnc-RPS6P3 Interacts with NP and NS1 Proteins

**Figure 3:**
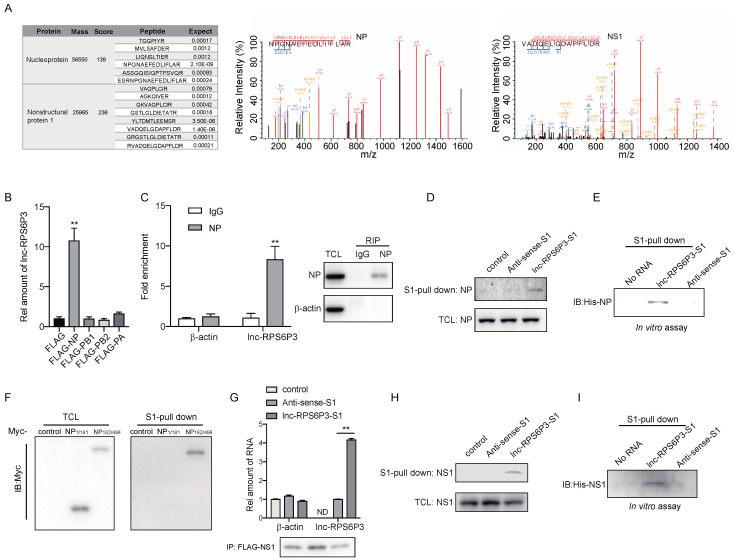
Lnc-RPS6P3 interacts with NP and NS1 proteins. (**A**) 293T cells were transfected with pLL3.7-AS-lnc-RPS6P3-S1 or pLL3.7-lnc-RPS6P3-S1, respectively, and infected with WSN. The cell lysates were incubated with streptavidin beads, and bound proteins were subjected to mass spectrometry (MS). NP and NS1 were identified as potential lnc-RPS6P3 interactors using mass spectrometry. (**B**,**C**) 293T cells were transfected with indicated plasmid, respectively, together with pLL3.7-lnc-RPS6P3 (**B**), or A549 cells were infected with WSN (**C**). The cell lysates were subjected to immunoprecipitation, followed by RT-qPCR, to determine the level of lnc-RPS6P3. (**D**) 293T cells were transfected with pLL3.7-S1, pLL3.7-AS-lnc-RPS6P3-S1 or pLL3.7-lnc-RPS6P3-S1, respectively, and infected with WSN. The cell lysates were subjected to S1-pulldown, followed by immunoblotting with indicated antibodies. (**E**) In vitro-transcribed S1 aptamer-tagged anti-sense or sense lnc-RPS6P3 were incubated with His-NP and subjected to S1-pull down. The bound NP were detected by immunoblotting with indicated antibodies. (**F**) 293T cells were transfected with the pcDNA-Myc-tagged-truncated NP as indicated. Cell lysates were incubated with in vitro-transcribed S1-lnc-RPS6P3, followed by S1-pulldown. The bound NP proteins were detected by immunoblotting. (**G**) 293T cells were co-transfected with pcDNA-FLAG-NS1 and pLL3.7-S1, pLL3.7-AS-lnc-RPS6P3-S1 or pLL3.7-lnc-RPS6P3-S1. Cell lysates were subjected to immunoprecipitation, followed by RT-qPCR to determine the level of lnc-RPS6P3. ND, not detected. (**H**) 293T cells were transfected with pLL3.7-S1, pLL3.7-AS-lnc-RPS6P3-S1 or pLL3.7-lnc-RPS6P3-S1, respectively, and infected with WSN. The cell lysates were subjected to S1-pulldown, followed by immunoblotting with indicated antibodies. (**I**) S1-tagged anti-sense or sense lnc-RPS6P3 were incubated with His-NS1 for S1-pulldown. The bound NS1 were detected by immunoblotting with indicated antibodies. Data are shown as the mean ± SD. ** *p* < 0.01; TCL, total cell lysis.

We next sought to explore the mechanisms of lnc-RPS6P3-inhibiting IAV replication. Recently, many lncRNAs have been reported to interact with proteins and regulate protein functions. To identify the protein(s) that interacted with lnc-RPS6P3, we performed RNA immunoprecipitation (RIP) and mass spectrometry (MS). The data showed that lnc-RPS6P3 can interact with NP and NS1 proteins (Figure 3A). To verify the interaction between lnc-RPS6P3 and the NP protein, we transfected 293T cells with pcDNA-FLAG-NP, pcDNA-FLAG-PB1, pcDNA-FLAG-PB2 or pcDNA-FLAG-PA, together with pLL3.7-lnc-RPS6P3, and collected the cell lysates for the RIP assay. The results indicated that lnc-RPS6P3 can interact with NP but not PB1, PB2 or PA (Figure 3B). Then, we confirmed that endogenous lnc-RPS6P3 interacted with NP by the RIP assay (Figure 3C) and the pulldown assay with S1-tagged lnc-RPS6P3 (Figure 3D). In addition, we carried out the pulldown assay with in vitro-transcribed S1-lnc-RPS6P3 or S1-antisense lnc-RPS6P3 as the control and purified His-tagged NP protein. The data showed that lnc-RPS6P3 can interact with the NP protein directly (Figure 3E). To further investigate whether the RNA-binding domain (RBD) of NP is responsible for interacting with lnc-RPS6P3, we generated truncated NP proteins and performed the RNA pulldown assay. Of note, the results revealed that lnc-RPS6P3 specifically interacted with the C-terminal (aa 182–498) of NP but not the N-terminal (aa 1–181) (the RBD) (Figure 3F). Similarly, we confirmed that NS1 interacted with lnc-RPS6P3 by the RIP assay (Figure 3G) and S1-tagged lnc-RPS6P3 with the pulldown assay (Figure 3H). In addition, we observed an interaction between the purified His-tagged NS1 protein and in vitro-transcribed S1-lnc-RPS6P3, indicating that lnc-RPS6P3 binds to NS1 directly (Figure 3I). Taken together, the above data confirmed that lnc-RPS6P3 can interact with NP and NS1 proteins directly.

### 3.4. Lnc-RPS6P3 Blocks vRNP Activity of Influenza A Virus by Interfering with NP Oligomerization

**Figure 4:**
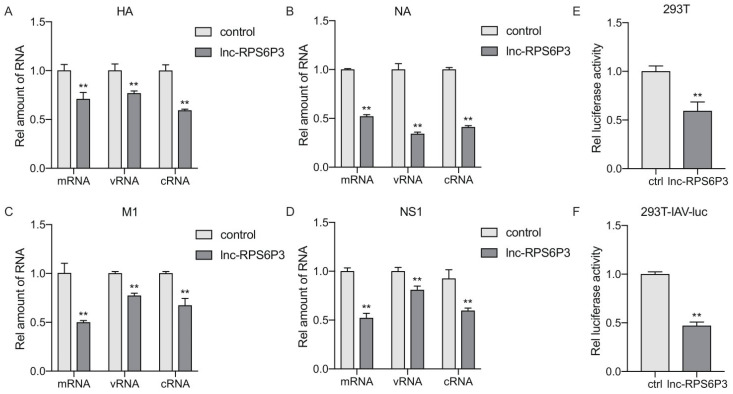
Lnc-RPS6P3 blocks the activity of vRNP. (**A**–**D**) 293T cells were transfected with pcDNA-PB2, pcDNA-PB1, pcDNA-PA, pCAGGS-NP and pPolI-WSN-HA (**A**), or pPolI-WSN-NA (**B**), pPolI-WSN-M (**C**) or pPolI-WSN-NS (**D**), together with pLL3.7-lnc-RPS6P3, for 36 h. The amount of the mRNA, vRNA and cRNA of HA, NA, M1 and NS1 were measured by RT-qPCR. (**E**) 293T cells were transfected with pcDNA-PB2, pcDNA-PB1, pcDNA-PA, pCAGGS-NP and pLL3.7-lnc-RPS6P3 with pHH21-vNS-Luc for 36 h. The cell lysates were collected and subjected to luciferase assay. (**F**) 293T-IAV-luc cells were transfected with pcDNA-PB2, pcDNA-PB1, pcDNA-PA, pCAGGS-NP and pLL3.7-lnc-RPS6P3 for 36 h. The cell lysates were harvested and subjected to luciferase assay. Data are shown as the mean ± SD. ** *p* < 0.01.

To gain insights into the mechanisms involved in lnc-RPS6P3-inhibiting IAV replication, we next investigated the effect of lnc-RPS6P3 on IAV RNA transcription and replication. We transfected the 293T cells with pcDNA-PB2, pcDNA-PB1, pcDNA-PA, pCAGGS-NP and pPolI-WSN-HA, or pPolI-WSN-NA, pPolI-WSN-M or pPolI-WSN-NS, together with pLL3.7-lnc-RPS6P3. The RT-qPCR results revealed that the level of mRNA, cRNA and vRNA of HA, NA, M1 and NS1 were significantly lower in lnc-RPS6P3-overexpressing cells than in control cells (Figure 4A–D). These data further suggested that lnc-RPS6P3 impaired the function of the IAV RdRp complex. To further confirm that lnc-RPS6P3 inhibits the vRNP activity directly, we transfected the 293T cell with pcDNA-PB2, pcDNA-PB1, pcDNA-PA and pCAGGS-NP, together with pLL3.7-lnc-RPS6P3 and pHH21-vNS-Luc. The data indicated that the luciferase activity was significantly lower in cells expressing lnc-RPS6P3 than in control cells (Figure 4E). We performed a similar experiment using 293T-IAV-luc cells, in which the IAV vRNP promoter was integrated into the genome of 293T cells, and we obtained a consistent result (Figure 4F). These results demonstrated that lnc-RPS6P3 blocks the vRNP activity of the influenza A virus.

**Figure 5:**
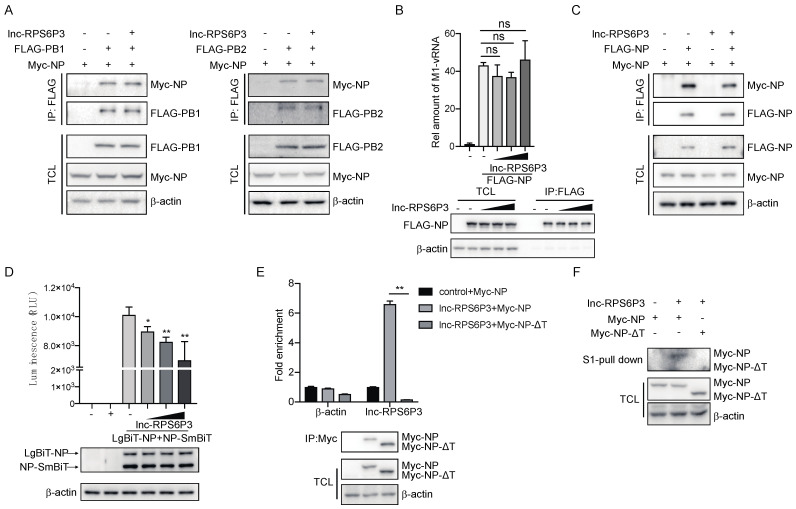
Lnc-RPS6P3 interferes with the self-oligomerization of NP protein. (**A**) 293T cells were transfected with indicated plasmid. The cell lysates were immunoprecipitated with anti-FLAG beads, followed by immunoblotting. (**B**) 293T cells were transfected with pcDNA-FLAG-NP, pPolI-WSN-M and different amount of pLL3.7-lnc-RPS6P3. The cell lysates were immunoprecipitated with anti-FLAG beads and subjected to RT-qPCR to quantify the amount of M1 vRNA. (**C**) 293T cells were transfected with indicated plasmid. The cell lysates were immunoprecipitated with anti-FLAG beads, followed by immunoblotting. (**D**) 293T cells were transfected with pLenti-EF1a-LgBiT-NP, pCDH-NP-SmBiT and different amount of pLL3.7-lnc-RPS6P3. The cell lysates were harvested for NanoLuc luciferase assays (**upper panel**). The protein levels of LgBiT-NP and NP-SmBiT in cells were detected by immunoblotting (**lower panel**). (**E**) 293T cells were transfected with pLL3.7-lnc-RPS6P3, together with pcDNA-Myc-NP or pcDNA-Myc-NP-ΔT (tail loop of NP (aa 402–428) was deleted). Cell lysates were immunoprecipitated with anti-Myc beads, followed by RT-qPCR to measure the level of lnc-RPS6P3. (**F**) 293T cells were transfected with indicated plasmid. The cell lysates were subjected to S1-pulldown, followed by immunoblotting indicated antibodies. Data are shown as the mean ± SD. * *p* < 0.05 and ** *p* < 0.01; ns, not significant; TCL, total cell lysis.

As NP can bind with PB1 and PB2 and form the vRNP complex, we examined whether lnc-RPS6P3 could interfere with the interaction between NP and PB1 or PB2. The data showed that overexpression of lnc-RPS6P3 did not affect the interaction of NP with PB1 or PB2 (Figure 5A). We then analyzed whether lnc-RPS6P3 would inhibit the binding of NP with viral RNA. The result indicated that lnc-RPS6P3 did not reduce the association of NP with vRNA (Figure 5B). NP oligomerization is critical for vRNP activity. Therefore, we checked whether lnc-RPS6P3 could influence oligomerization of the NP protein by co-immunoprecipitation assays. The data indicated that overexpression of lnc-RPS6P3 obviously reduced the interaction of FLAG-NP and Myc-NP (Figure 5C). Furthermore, we generated pLenti-EF1a-LgBiT-NP and pCDH-NP-SmBiT and analyzed the association of the NP protein by using the Nanobit–luciferase assay. The data showed that the Nanobit–luciferase activity was significantly reduced in lnc-RPS6P3-overexpressing cells compared with control cells (Figure 5D). These results demonstrated that lnc-RPS6P3 can bind with the NP protein and inhibit its oligomerization. Given that the tail loop (aa 402–428) of NP is essential for its oligomerization, we postulated that lnc-RPS6P3 might specifically bind to this region to prevent oligomerization of NP. To test this, we constructed the tail loop-deleted NP (NP-ΔT) plasmid and then performed the RIP assay and the pulldown assay. The result revealed that lnc-RPS6P3 binds to the wild-type (WT) NP but not NP-ΔT (Figure 5E,F). Collectively, these data demonstrate that lnc-RPS6P3 blocked vRNP activity by suppressing oligomerization of the NP protein.

### 3.5. Lnc-RPS6P3 Reduces the Inhibitory Effect of NS1 on IFN-β Activation by Decreasing the Interaction of NS1 with RIG-I and TRIM25

**Figure 6:**
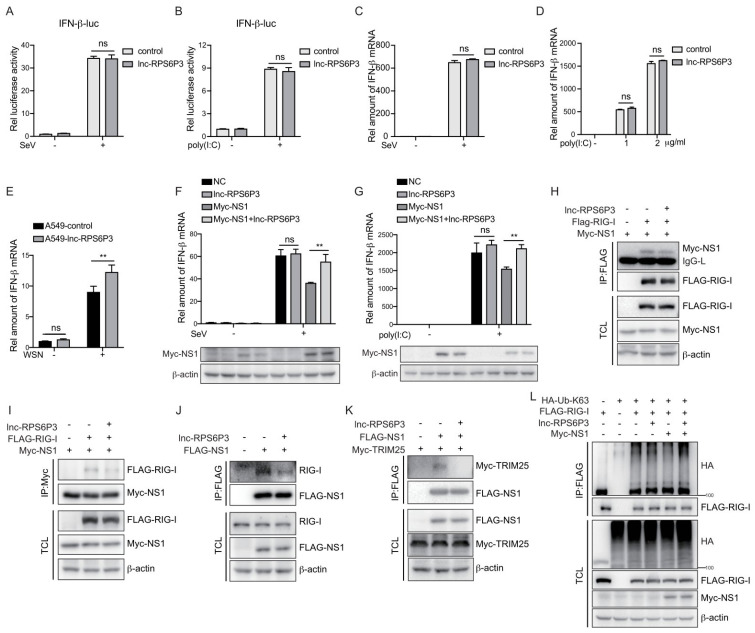
Lnc-RPS6P3 reduced the inhibitory effect of NS1 on RIG-I signaling pathway. (**A**,**B**) 293T cells were transfected with pGL4-IFN-β-luc, together with control or pLL3.7-lnc-RPS6P3, for 24 h. Then, the cells were infected with SeV (**A**) or transfected with poly(I:C) (**B**) for 12 h. The cell lysates were harvested for luciferase assays. (**C**,**D**) 293T cells were transfected with control or pLL3.7-lnc-RPS6P3 for 24 h. Then, the cells were infected with SeV (**C**) or transfected with poly(I:C) (**D**) for 12 h. The amounts of IFN-β mRNA were measured by RT-qPCR. (**E**) A549 cells were infected with lentivirus-expressing lnc-RPS6P3 or control lentivirus. The cells were then infected with or without WSN at MOI of 0.1 for 12 h. The amounts of IFN-β mRNA were measured by RT-qPCR. (**F**,**G**) 293T cells were transfected individually or in combination with pLL3.7-lnc-RPS6P3 and pCMV-Myc-NS1 and then infected with SeV for 6 h (**F**) or transfected with poly(I:C) for 12 h (**G**). The amounts of IFN-β mRNA were measured by RT-qPCR. (**H**–**L**) 293T cells were transfected with indicated plasmid. Cell lysates were subjected to immunoprecipitation and immunoblotting with indicated antibodies. Data are shown as the mean ± SD. ** *p* < 0.01; ns, not significant; TCL, total cell lysis.

To investigate the role of lnc-RPS6P3 in a host anti-IAV innate immune response, we first examined whether lnc-RPS6P3 can promote IFN-β activation. As shown in Figure 6A,B, lnc-RPS6P3 did not affect the promoter activity of IFN-β upon stimulation of the Sendai virus infection and poly(I:C) treatment. Consistently, lnc-RPS6P3 did not influence the mRNA level of IFN-β upon stimulation of the Sendai virus infection and poly(I:C) treatment (Figure 6C,D). However, lnc-RPS6P3 promoted the mRNA level of IFN-β upon IAV infection (Figure 6E), implying that lnc-RPS6P3 may antagonize the inhibitory effect of NS1 upon IFN-β activation. To address this question, we transfected with pLL3.7-lnc-RPS6P3 and pCMV-Myc-NS1 in 293T cells and then infected with the Sendai virus, or transfected with poly(I:C), and examined the level of IFN-β mRNA. The results showed that the NS1 protein inhibited the expression of IFN-β, while lnc-RPS6P3 could partially restore the level of IFN-β mRNA (Figure 6F,G). It is reported that the NS1 interacts with RIG-I and TRIM25 and inhibits the RIG-I-mediated induction of IFN-β. Therefore, we postulated that the lnc-RPS6P3 may reduce the inhibitory effect of NS1 on the functions of RIG-I and TRIM25, which promotes the induction of IFN-β. We investigated whether lnc-RPS6P3 could interfere with the interaction between NS1 and RIG-I or TRIM25. The data showed that overexpression of lnc-RPS6P3 inhibited the interaction of NS1 with RIG-I (Figure 6H–J) and TRIM25 (Figure 6K). Furthermore, we examined whether lnc-RPS6P3 could affect the RIG-I ubiquitination, which indicates the activation of RIG-I. The data showed that NS1 inhibited the ubiquitination of RIG-I, while lnc-RPS6P3 rescued the ubiquitination of RIG-I (Figure 6L). Together, these results indicated that lnc-RPS6P3 attenuated the inhibitory effect of NS1 on IFN-β activation by reducing the interaction of NS1 with RIG-I and TRIM25.

## 4. Discussion

It has been reported that lncRNAs exert various regulatory activities by interacting with DNA, RNA or proteins during viral infection. On one hand, lncRNAs regulate viral replication by direct interaction with viral proteins. For example, lncRNA IPAN associates with the IAV PB1 protein to form the IPAN/PB1 complex and thus further stabilizes the PB1 protein, which enables efficient viral RNA synthesis [32]. LncRNA-PAAN interacts with the viral PA protein to promote the assembly of viral RNA polymerase, thus warranting efficient viral replication [33]. On the other hand, lncRNAs can be involved in host antiviral innate immunity, influencing viral replication. For instance, lncRNA-ISG20 enhanced ISG20 expression to inhibits influenza A virus replication via acting as a miR-326 sponge [26]. LncRNA-MxA inhibited the transcription of IFN-β to promote influenza A virus replication via the formation of RNA–DNA triplexes [29]. In addition, lncRNA-GM promotes an antiviral innate response by binding to glutathione S-transferase M1 (GSTM1) and blocking its interaction with the kinase TBK1 to enhance TBK1 activity and downstream signaling pathway [34]. However, in this study, we found that lnc-RPS6P3 displayed functions to inhibit viral replication in both direct and indirect manners. It can not only suppress vRNP activity by inhibiting oligomerization of NP but also induce transcription of IFN-β to improve host antiviral innate immunity by interfering the interaction of NS1 with RIG-I. Our finding suggests that lncRNAs could play multiple roles during viral infection.

We have demonstrated that lnc-RPS6P3 associates with both IAV NP and NS1 to regulate their functions. We also found, through mass spectrometry, that lnc-RPS6P3 associates with host proteins. These proteins include cellular metabolic enzymes involved in metabolism pathways, such as PCCB, PCCA and ACC1, and cytoskeletal proteins. It has been reported that inhibitors of ACC (acetyl-CoA carboxylase) significantly inhibited influenza A virus replication [35]. We speculate that lnc-RPS6P3 may affect the enzymatic activity of ACC1 to decrease influenza virus replication, which needs further investigation. The cellular cytoskeleton plays a vital role in the lifecycle of the influenza virus [36]. Researchers found that IAV vRNP is transported from the nucleus into the cytoplasm in a nuclear F-actin-dependent manner, while IAV infection significantly induced the formation of actin microfilaments (F-actin) in the nucleus [37]. Dynein, myosin II, MTs and actin were also involved in the uncoating of IAV after it is entered, by endocytosis [38]. Our data showed that lnc-RPS6P3 can interact with the cytoskeleton, such as MYH9, Vimentin and Plectin. A previous study demonstrated that LncRNA MAFG-AS1 promoted ATPase activity of MYH9 by interacting with it [39]. We postulated that lnc-RPS6P3 may also regulate the activity of cytoskeleton proteins and consequently influence the IAV replication. However, whether lnc-RPS6P3 could inhibit influenza virus replication by regulating other pathways, such as lipid metabolism and the cytoskeleton, needs further elucidated.

Human lnc-RPS6P3 is a pseudogene lncRNA. Studies have shown that pseudogene-derived lncRNAs play important roles in different ways. For example, lncRNA Olfr29-ps1 directly sponged miR-214-3p to downregulate miR-214-3p and thus promoted the immunosuppressive function and differentiation of monocytic MDSCs [40]. The pseudogene-derived PTTG3P formed an RNA-protein complex with ILF3 to maintain MAP2K6 and E2F1 mRNA stability and promoted NSCLC cell proliferation and tumorigenesis [41]. In addition, humans and several primates possess the PTEN pseudogene (PTENP1) that gives rise to long non-coding RNA lncPTENP1-S, which plays a pro-oncogenic role in GBM cells by upregulating the expression of cancer stem cell markers and decreasing cell adhesion [42]. Together with our finding, we believe that pseudogene-derived lncRNAs are emerging as interesting elements capable of being potentially functional.

Here, we demonstrated that lnc-RPS6P3 hindered the inhibitory effect of NS1 on the RIG-I pathway. In fact, in addition to IAV, other viruses have also evolved different strategies to escape the host antiviral immune response. For instance, DENV NS4a inhibited the interaction between RIG-I and MAVS, resulting in blocking the RIG-I/mitochondrial antiviral signaling (MAVS) pathway and IFN-β induction [43]. HIV-associated Vpr and Vif disrupted TBK1 trans-autophosphorylation via binding to TBK1 and inhibited subsequent IRF3 phosphorylation and type I/III IFN gene expression [44]. It will be worthwhile to investigate whether lnc-RPS6P3 can bind other viral proteins to antagonize their functions and thus restore antiviral innate immunity.

Collectively, these data demonstrate that lnc-RPS6P3 as a pseudogene-derived lncRNA is induced by viral infection and the IFN signaling pathway and binds to the viral proteins NP and NS1. Upon viral infection, lnc-RPS6P3 inhibits oligomerization of NP, leading to a reduction in vRNP activity and interferes the binding of NS1 to RIG-I, leading to increased K63 ubiquitination of RIG-I, which activates the antiviral innate response and inhibits influenza virus replication.

## 5. Conclusions

To summarize, we identified that pseudogene-derived lnc-RPS6P3 is up-regulated during IAV infection and is also an interferon-stimulated gene. We demonstrated that lnc-RPS6P3 can inhibit IAV replication. Our studies revealed the detailed mechanism of how lnc-RPS6P3 negatively regulates IAV replication. Our findings provide the evidence that pseudogene-derived lncRNA could play an important role during viral infection and the antiviral immune response.

## Data Availability

All the data generated during the current study are included in the manuscript.
